# Prognostic and clinicopathological significance of circulating tumor cells detected by RT-PCR in non-metastatic colorectal cancer: a meta-analysis and systematic review

**DOI:** 10.1186/s12885-017-3704-8

**Published:** 2017-11-07

**Authors:** Chaogang Yang, Kun Zou, Liang Zheng, Bin Xiong

**Affiliations:** 1 Department of Gastrointestinal Surgery & Department of Gastric and Colorectal Surgical Oncology, Zhongnan Hospital of Wuhan University; Hubei Key Laboratory of Tumor Biological Behaviors & Hubei Cancer Clinical Study Center, No.169 Donghu Road, Wuchang District, Wuhan, 430071 China; 2 Department of Oncology, Central Hospital of Wuhan, No.16 Gusaoshu Road, Jianghan District, Wuhan, 430014 China

**Keywords:** Circulating tumor cells, Non-metastatic colorectal cancer, RT-PCR, Prognosis, Meta-analysis

## Abstract

**Background:**

Circulating tumor cells (CTCs) have been accepted as a prognostic marker in patients with metastatic colorectal cancer (mCRC, UICC stage IV). However, the prognostic value of CTCs in patients with non-metastatic colorectal cancer (non-mCRC, UICC stage I-III) still remains in dispute. A meta-analysis was performed to investigate the prognostic significance of CTCs detected by the RT-PCR method in patients diagnosed with non-mCRC patients.

**Methods:**

A comprehensive literature search for relevant articles was performed in the EmBase, PubMed, Ovid, Web of Science, Cochrane library and Google Scholar databases. The studies were selected according to predetermined inclusion/exclusion criteria. Using the random-effects model of Stata software, version12.0 (2011) (Stata Corp, College Station, TX, USA), to conduct the meta-analysis, and the hazard ratio (HR), risk ratio (RR) and their 95% confidence intervals (95% CIs) were regarded as the effect measures. Subgroup analyses and meta-regression were also conducted to clarify the heterogeneity.

**Results:**

Twelve eligible studies, containing 2363 patients with non-mCRC, were suitable for final analyses. The results showed that the overall survival (OS) (HR = 3.07, 95% CI: [2.05–4.624], *P* < 0.001; I^2^ = 55.7%, *P* = 0.008) and disease-free survival (DFS) (HR = 2.58, 95% CI: [2.00–3.32], *P* < 0.001; I^2^ = 34.0%, *P* = 0.085) were poorer in patients with CTC-positive, regardless of the sampling time, adjuvant therapy and TNM stage. CTC-positive was also significantly associated with regional lymph nodes (RLNs) metastasis (RR = 1.62, 95% CI: [1.17–2.23], *P* = 0.003; I^2^ = 74.6%, *P*<0.001), depth of infiltration (RR = 1.41, 95% CI: [1.03–1.92], *P* = 0.03; I^2^ = 38.3%, *P* = 0.136), vascular invasion (RR = 1.66, 95% CI: [1.17–2.36], *P* = 0.004; I^2^ = 46.0%, *P* = 0.135), tumor grade (RR = 1.19, 95% CI: [1.02–1.40], *P* = 0.029; I^2^ = 0%, *P* = 0.821) and tumor-node-metastasis (TNM) stage(I, II versus III) (RR = 0.76, 95% CI 0.71–0.81, *P* < 0.001; I^2^ = 0%, *P* = 0.717). However, there was no significant relationship between CTC-positive and tumor size (RR = 1.08, 95% CI: [0.94–1.24], *P* = 0.30; I^2^ = 0%, *P* = 0.528).

**Conclusions:**

Detection of CTCs by RT-PCR method has prognostic value for non-mCRC patients, and CTC-positive was associated with poor prognosis and poor clinicopathological prognostic factors. However, the prognostic value of CTCs supports the use of CTCs as an indicator of metastatic disease prior to the current classification of mCRC meaning it is detectable by CT/MRI.

## Background

Colorectal cancer (CRC) is the third most commonly diagnosed cancer and the fourth leading cause of cancer-related death [1]. In China, CRC is ranked fourth in morbidity and mortality among the gastrointestinal cancers [2]. Due to the difficulties of early diagnosis, a large proportion of patients with CRC are undiagnosed until an advanced stage. Due to the continuous improvement of the treatment methods, decreasing CRC mortality rates have been observed in a large number of countries worldwide [3], especially for the patients with non-metastatic colorectal cancer (non-mCRC, UICC I-III). Unfortunately, the 5-year overall survival (OS) of non-mCRC patients is still low and approximately 25–50% of patients with stage II-III CRC will experience recurrence or distant metastasis after comprehensive treatment [4], which is the main reason for studying the prognosis in those patients. The mechanisms of recurrence and metastasis of CRC are very complicated and remains unclear. Recurrence and metastasis may involve series of cell biological behaviors, including circulating tumor cells (CTCs), which have been gradually recognized to play an important role in the process of distant metastasis, according to the “seed and soil theory” [5].

CTCs, which were defined as the “break away” cancer cells in the peripheral blood (PB) of cancer patients, were firstly proposed by Ashworth in1869 [6] and further demonstrated by Engell in 1955 [7]. These cells, which shed intermittently from the solid tumors, circulate in the bloodstream, and arrive at different positions, are the main cause of distant metastases [8]. However, the lower concentration of PB in the solid tumors, which are confined to local growth [9, 10], makes it difficult to detect in early CRC. During the past few decades, with a variety of highly sensitive and specific diagnostic approaches including reverse transcriptase-polymerase chain reaction (RT-PCR), immunocytochemistry, flow cytometry, and the CellSearch system, the efficiency of detecting CTCs is increasing gradually. Encouraging results from numerous studies have demonstrated that the presence of CTCs was significantly associated with poor prognosis of CRC patients. However, most large-scale data were collected from patients with mCRC [11, 12], there were only limited data on the significance of CTC in patients with non-mCRC. In those studies, the diagnostic method used to detect CTCs was predominantly the CellSearch system [13–15], which is the first and only method approved by the US Food and Drug Administration (FDA) for evaluating the prognosis of CRC patients [16]. However, while there are advantages of high specificity and reproducibility for CTC detection, as a semi-automated system, CellSearch has the disadvantages of moderate sensitivity and subjective verification. Compared to CellSearch, RT-PCR has higher sensitivity and is more objective for detection of CTCs [17, 18]. Therefore, it has also been widely used for the detection of CTCs for non-mCRC patients, and the clinical utility has been demonstrated in several studies. Shimada et al. reported that CTCs detection with the RT-PCR method was correlated with tumor metastasis and prognosis [19]. However, Kust et al. showed that CTCs detected with RT-PCR had unfavorable prognostic significance for non-mCRC patients [20]. Therefore, the prognostic role of CTC detection with RT-PCR in non-mCRC is still controversial.

We performed a pooled analysis of published studies to quantitatively and comprehensively summarize the prognostic relevance of CTCs detected by RT-PCR in patients with non-mCRC.

## Methods

### Search strategy

A literature search for relevant studies was performed systematically from the EmBase, PubMed, Ovid, Web of Science, Cochrane library and Google Scholar database with key words “colorectal cancer”, “circulating tumor cells” or “CTCs” and “polymerase chain reaction or PCR” by two researchers (CG Yang and K Zou) independently (up to July, 2016). No time restriction was imposed. In order to prevent missing relevant studies, “related articles” function of PubMed and Google Scholar were used to identify other potentially relevant publications.

### Inclusion and exclusion criteria

The inclusion criteria for our meta-analysis were: (1) investigated the clinicopathological or prognostic significance of CTC detection in non-mCRC patients; (2) used any form of RT-PCR for detecting CTCs; (3) hazard ratio (HR) or a risk ratio(RR) with a 95% confidence interval (95% CI) of OS or/and disease-free survival (DFS) reported in the study or had sufficient data to calculate; (4) collected the samples from PB. Exclusion criteria were: (1) studies including mCRC patients; (2) the number of patients was less than 20. (3) exclusion of letters, reviews, and articles published with non-English language. (4) the study was redundant, based on the same database or patient population as an included study. To avoid the inclusion of redundant studies, all the included studies were checked carefully, including their authors, organizations, accrual period, and population of patients.

### Data extraction and quality assessment

Two reviewers (CG Yang and K Zou) evaluated the quality of the included studies and extracted data independently. The following information was collected: first author, year of publication, country, characteristics of the study population (number, sex and age), TNM stage (UICC), detection markers, adjuvant therapy, sampling time (pre/intra/post-operation), rate of CTC positivity rate, follow-up period, the HR and their associated standard errors on prognostic outcomes (OS or/and DFS). If the HRs and its 95% CI were not directly provided in the original articles, we used the method designed by Jayne F. Tierney [21] to calculate them from the available data. In addition, when HRs were presented by both univariate and multivariate analyses, the latter ones were preferable because multivariate analyses also considered possible confounding of exposure effects [22]. The quality assessment was based on the Newcastle-Ottawa Scale (NOS) criteria, which is recommended by the Cochrane Library for the cohort study, score 5–9 is considered as high quality and 1–4 is low quality [23]. The results of quality assessment and data extraction were confirmed by two reviewers. Any disagreements about data extraction and quality assessment were resolved by comprehensive discussion and were checked by the third investigator.

### Statistical analysis

Statistical analyses were implemented with Stata software, version 12.0 (2011) (Stata Corp, College Station, TX, USA). The RR and HR were regarded as effect measures for summarizing the clinicopathological and prognostic significance of CTCs detected by RT-PCR in non-mCRC. By convention, a HR >1 indicates a poorer prognosis in the CTC-positive group in contrast with negative group and a RR > 1 implies CTC-positive be associated with a parameter. All statistical values were reported with 95% confidence intervals (95% CIs) and *P* value < 0.05 was considered statistically significant. To retain maximum information, we added additional information into included study from original authors or excluded studies if the included and excluded studies were based on the same patients’ population and some information of interest was reported in the excluded studies but not in the included studies. All relevant studies were included in the overall analysis. Subgroup analyses were performed based on the sampling time (pre/intra/post-OP), TNM stage (II/III), adjuvant therapy (without/post-OP chemotherapy) and detection markers (single/multiple). All data analyses used a random effects model, because it provided more conservative estimates and more tailored to multicenter studies in which heterogeneity was usually present [24]. The Cochrane’s Q statistic and I^2^ statistic were applied to evaluate the heterogeneity among studies. *P* value < 0.01 for the Q statistic and/or I^2^ > 50% were considered significant heterogeneity [25]. The I^2^ value indicated the degree of heterogeneity. Potential heterogeneity between-study was illustrated by forest plots. If necessary, meta-regression was performed to explore the potential source of heterogeneity. Lastly, we evaluated potential publication bias by a funnel plot, which was further validated by the Egger [26] and Begg’s test [27].

## Results

### Baseline characteristics of the eligible studies

**Fig. 1:**
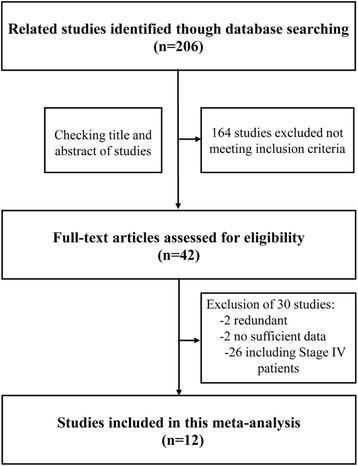
Flow chart showing the selection process for the included studies

Initially, 206 relevant studies were identified in the systematic literature search process. By checking the titles and abstracts, 164 studies were excluded and 42 potential studies were retrieved. An additional 30 studies were then excluded after they were fully reviewed because they lacked sufficient data (2 studies), were redundant (2 studies), or included stage IV patients (26 studies). Finally, 12 studies were yielded as meeting our inclusion criteria and were eligible for our meta-analysis (Fig. 1).

Twelve eligible studies, including 23 sets of data, contained 2363 patients with non-mCRC [19, 20, 28–37]. The studies were conducted in seven countries (Australia, China, Croatia, Germany, Japan, Spain and the UK) and were published between 2002 and 2016. All studies detected tumor cells from PB with the molecular detection method (PCR, RT-PCR, or RT followed by quantitative PCR). Table 1 summarizes the main baseline characteristics and study design variables. The quality of the eligible cohort studies was assessed with NOS and is summarized in Table 2.

**Table 1:** Baseline characteristics of the included studies

Article	Country	Number (M/F)^a^	Age Mean ± SD^b^/Median (range) (y^c^)	TS^d^	Markers	Adjuvant therapy	ST^e^	Rate (+)^f^	OM^g^	HR^h^ estimate	CS^i^	MA^j^
Kust 2016 [33]	Croatia	82(49/33)	Mean:66 ± 9.6	I-III	CK20	Post-OP^k^ CT^l^ for III and part of II	Pre-OP	22 (26.83%)	OS^m^ DFS^n^	Data extrapolated	Yes	No
Liu 2013 [28]	China	92(60/32)	Mean:66 ± 9.6	I-III	CK20	_	Pre-OP	31 (25%)	OS DFS	Reported in text	Yes	Yes
Liu 2013 (1) [28]	China	41(NR)	NR^o^	II	CK20	Without	_	NR	OS DFS	Reported in text	Yes	Yes
Liu 2013 (2) [28]	China	51(NR)	NR	III	CK20	Post-OP CT	_	NR	OS DFS	Reported in text	Yes	Yes
Yokobori (1) 2013 [29]	Japan	Training: 151(86/65)	Mean:66.76 ± 11.02	II	PLS3	Without	Pre-OP	33 (21.85%)	OS DFS	Reported in text	Yes	Yes
Yokobori (2) 2013 [29]	Japan	Training: 131(75/56)	Mean:66.76 ± 11.02	III	PLS3	Post-OP CT	Pre-OP	38 (29%)	OS DFS	Reported in text	Yes	Yes
Yokobori (3) 2013 [29]	Japan	Validation: 158(96/62)	Mean:67.51 ± 11.08	II	PLS3	Without	Pre-OP	35 (22.15%)	OS DFS	Reported in text	Yes	Yes
Yokobori (4) 2013 [29]	Japan	Validation: 103(63/40)	Mean:67.51 ± 11.08	III	PLS3	Post-OP CT	Pre-OP	30 (29.12%)	OS DFS	Reported in text	Yes	Yes
Shimada (1) 2012 [19]	Japan	111(60/51)	Median:68(27–82)	II	CEA, CK19, CK20, CD133	Without	Post-OP	63 (56.76%)	OS DFS	Reported in text	Yes	Yes
Shimada (2) 2012 [19]	Japan	86(47/39)	Median:68(27–82)	III	CEA, CK19, CK20, CD133	Post-OP CT	Post-OP	61 (70.93%)	OS DFS	Reported in text	Yes	Yes
Iinuma (1) 2011 [30]	Japan	Training: 420(224/196)	Mean:66.0 ± 12.4	I-III	CEA, CK19, CK20, CD133	_	Post-OP	106(25.24%)	OS DFS	Reported in text	Yes	Yes
Iinuma (1–1) 2011 [30]	Japan	Training: 176(NR)	NR	II	CEA, CK19, CK20, CD133	Without	_	NR	OS DFS	Reported in text	Yes	Yes
Iinuma (1–2) 2011 [30]	Japan	Training:150(NR)	NR	III	CEA, CK19, CK20, CD133	Post-OP CT	_	NR	OS DFS	Reported in text	Yes	Yes
Iinuma (2) 2011 [30]	Japan	Validation: 315(175/140)	Mean:66.0 ± 12.4	I-III	CEA, CK19, CK20, CD133	_	Post-OP	75(23.81%)	OS DFS	Reported in text	Yes	Yes
Iinuma (2–1) 2011 [30]	Japan	Validation: 143(NR)	NR	II	CEA, CK19, CK20, CD133	Without	_	NR	OS DFS	Reported in text	Yes	Yes
Iinuma (2–2) 2011 [30]	Japan	Validation: 97(NR)	NR	III	CEA, CK19, CK20, CD133	Post-OP CT	_	NR	OS DFS	Reported in text	Yes	Yes
Uen 2008 [31]	China	438(234/204)	NR	I-III	CK19, CK20, CEA, hTERT	Post-OP CT for III and part of II	Post-OP (1 W^p^)	137 (31.27%)	DFS	Reported in text	Yes	Yes
Barreto 2007 [32]	UK	113(NR)	Mean:67	I-III	CEA, CK20	Post-OP CT for III and part of II	Post-OP (24 h^q^)	34 (30.09%)	DFS	Reported in text	Yes	Yes
Koch 2006 [33]	Germany	90(59/31)	Mean:66.1	II	CK20	Post-OP CT for rectal cancer	Post-OP	28 (31.1%)	OS DFS	Reported in text	Yes	No
Lloyd 2006 [34]	Australia	125 (74/51)	Median:74 (43–95)	I-II	CK20, CEA, EphB4, LAMγ2, MAT	Without	Pre-OP	5 (4%)	DFS	Data extrapolated	Yes	No
Sadahrio 2005 [35]	Japan	100(NR)	NR	I-III	CEA	NR	Intra-OP	39 (39%)	OS DFS	Data extrapolated	Yes	Yes
Bessa 2003 [36]	Spain	66 (23/43)	Median:73	I-III	CEA	Post-OP CT for III and part of II	Post-OP	36 (54.55%)	OS DFS	Reported in text	Yes	Yes
Ito 2002 [37]	Japan	99(62/37)	NR	I-III	CEA	NR	Post-OP	26 (26.26%)	DFS	Data extrapolated	Yes	No

NOTE: ^a^ *M/F* Male/female, ^b^ *SD* Standard deviation, ^c^ *Y* Year, ^d^ *TS* Tumor stage (UICC), ^e^ *ST* Sampling time, ^f^ *Rate (+)* Rate of CTCs-positive patients, ^g^ *OM* Outcome measured, ^h^ *HR* Hazard ratio, ^i^ *CS* Curative surgery, ^j^ *MA* Multivariance analysis, ^k^ *OP* Operation, ^l^ *CT* Chemotherapy, ^m^ *OS* Overall survival, ^n^ *DFS* Disease-free survival, ^o^ *NR* Not reported, ^p^ *W* Week, ^q^ *h* hour

**Table 2:** The assessment of the risk of bias in each Cohort study using the Newcastle-Ottawa Scale

Study	Selection (0–4)				Comparability (0–2)		Outcome (0–3)		Total	
	REC	SNEC	AE	DO	SC	AF	AO	FU	AFU	
Kust 2016 [20]	0	1	1	1	0	0	0	1	0	4
Liu 2013 [28]	0	1	1	1	0	0	1	1	0	5
Yokobori 2013 [29]	1	1	1	1	0	0	1	1	0	6
Shimada 2012 [19]	1	1	1	1	0	0	1	1	1	7
Iimuna 2011 [30]	0	1	1	1	0	0	1	1	0	5
Uen 2008 [31]	0	1	1	1	0	0	1	1	0	5
Barreto 2007 [32]	0	0	1	1	0	0	1	1	0	4
Koch 2006 [33]	0	1	1	1	0	0	1	0	0	4
Lloyd 2006 [34]	0	0	1	1	0	0	1	0	0	3
Sadahrio 2005 [35]	1	1	1	1	0	0	1	1	1	7
Bessa 2003 [36]	0	1	1	1	0	0	1	1	0	5
Ito 2002 [37]	0	1	1	1	0	0	1	1	0	5

NOTE: *REC* representativeness of the exposed cohort, *SNEC* selection of the non-exposed cohort, *AE* ascertainment of exposure; *DO*: demonstration that outcome of interest was not present at start of study, *SC* study controls for age, sex, *AF* study controls for any additional factors (chemoradiotherapy, curative resection), *AO* assessment of outcome, *FU* follow-up long enough (36 M) for outcomes to occur, *AFU* adequacy of follow-up of cohorts (≥90%).Total: the points of each study

### Effects of CTCs on OS and DFS for non-mCRC patients

**Fig. 2:**
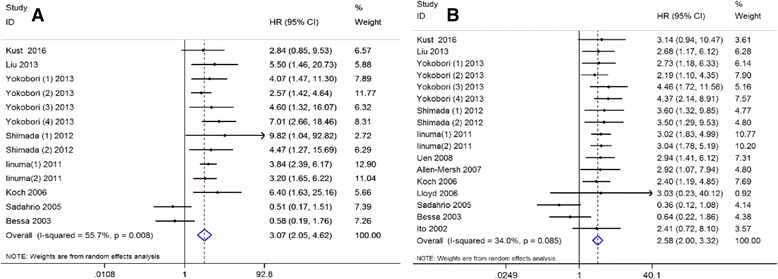
Summary estimates of hazard ratio for overall survival and disease-free survival of patients with CTC positivity. **a** overall survival; **b** disease-free survival

**Fig. 3:**
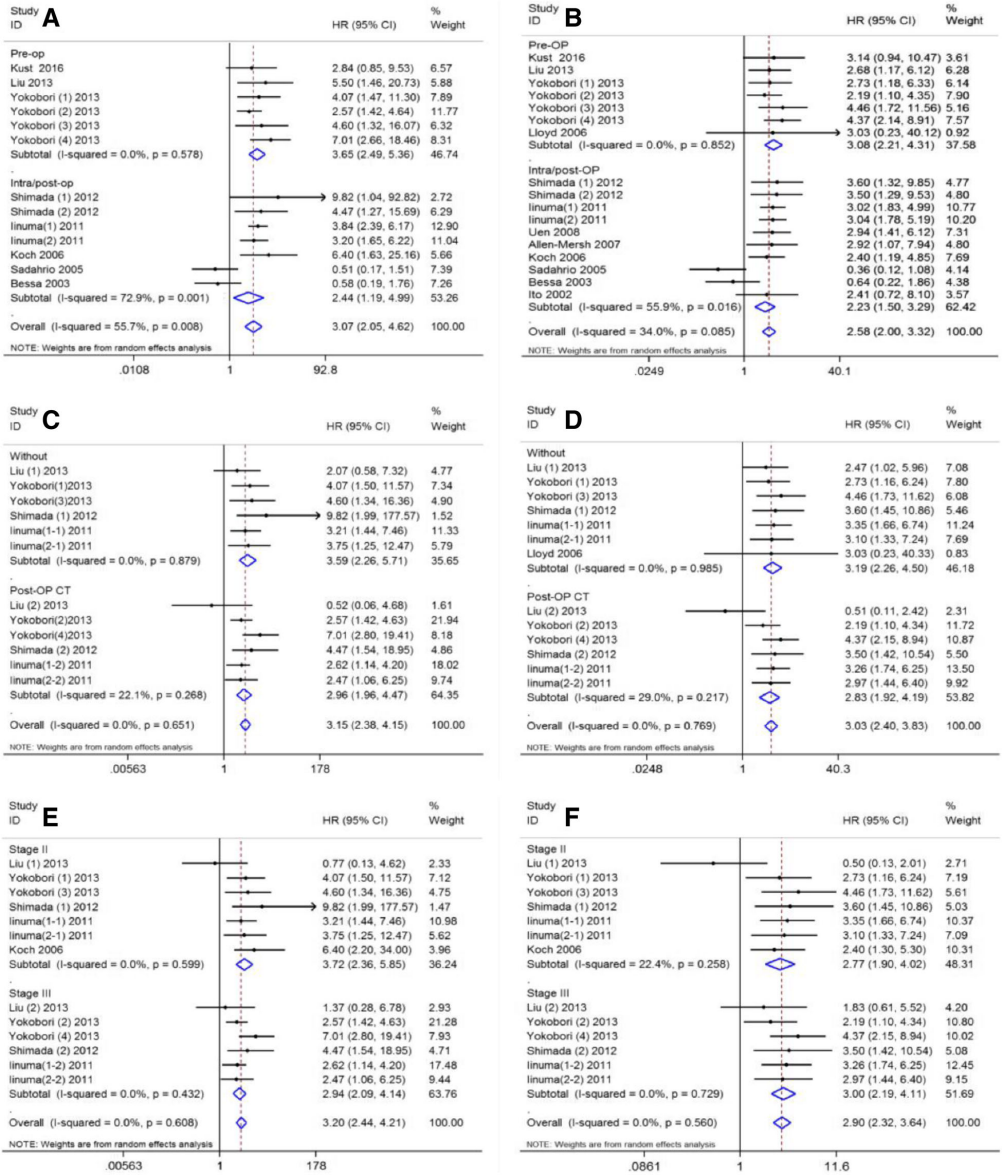
Subgroup analyses. **a**&**b** Pre-operation and intra/post-operation on overall survival and disease-free survival, respectively; **c**&**d** Without and with post-chemotherapy on overall survival and disease-free survival, respectively; **e**&**f** Stage II and stage III on overall survival and disease-free survival, respectively

Data on OS were available in 13 sets of data included in eight studies [19, 20, 28–30, 33, 35, 36]. The pooled analysis showed CTC-positive was significantly associated with a poor OS (HR = 3.07, 95% CI: [2.05–4.624], *P* < 0.001), with significant between-study heterogeneity (I^2^ = 55.7%, *P* = 0.008; Fig. 2a) in non-mCRC patients. Seventeen sets of data included in all enrolled studies contained the data on DFS [19, 20, 28–37]; the pooled analysis indicated CTC-positive was also associated with a significantly decreased DFS (HR = 2.58, 95% CI: [2.00–3.32], *P *< 0.001) with no between-study heterogeneity (I^2^ = 34.0%, *P* = 0.085; Fig.  2b). To further investigate the effect of CTCs detection on the prognosis of non-mCRC patients under different conditions, subgroup analyses were performed based on different sampling time (pre-OP and intra/post-OP), TNM stage (II/III) and adjuvant therapy status (without/post-OP chemotherapy). The results demonstrated CTC-positive was significantly associated with poor OS (HR = 3.65, 95% CI: [2.49–5.36], *P* < 0.001; HR = 2.44, 95% CI: [1.19–4.99], *P* = 0.015; Fig. 3a) and DFS (HR = 3.08, 95% CI: [2.21–4.31], *P* < 0.001; HR = 2.23, 95% CI: [1.50–3.29], *P* < 0.001; Fig. 3b) in non-mCRC patients, regardless of pre-OP or intra/post-OP sample collection. Furthermore, due to the limited number of studies on about neoadjuvant radiotherapy or/and chemotherapy and post-OP adjuvant radiotherapy in the included studies, we conducted a subgroup analysis to evaluate to prognostic value of CTCs in patients who did and did not receive post-operative chemotherapy. The results showed no difference between these two groups (OS, HR = 2.96, 95% CI: [1.96–4.47], *P* < 0.001; HR = 3.59, 95% CI: [2.26–5.71], *P* = 0.015; Fig. 3c. DFS, HR = 2.83, 95% CI: [1.92–4.19], *P* < 0.001; HR = 3.19, 95% CI: [2.26–4.50], *P* < 0.001; Fig. 3d). For TNM stage, subgroup analyses were only performed to explore the prognostic value of CTCs for stage II and III CRC patients; the results demonstrated that CTC-positive was significantly associated with poor OS (HR = 3.72, 95% CI: [2.36–5.85], *P* < 0.001; HR = 2.94, 95% CI: [2.09–4.14], *P* < 0.001; Fig. 3e) and DFS (HR = 2.77, 95% CI: [1.90–4.02], *P* < 0.001; HR = 3.00, 95% CI: [2.19–4.11], *P* < 0.001; Fig. 3f) for both stage II and III CRC patients.

### Association between CTCs and clinicopathological parameters

**Fig. 4:**
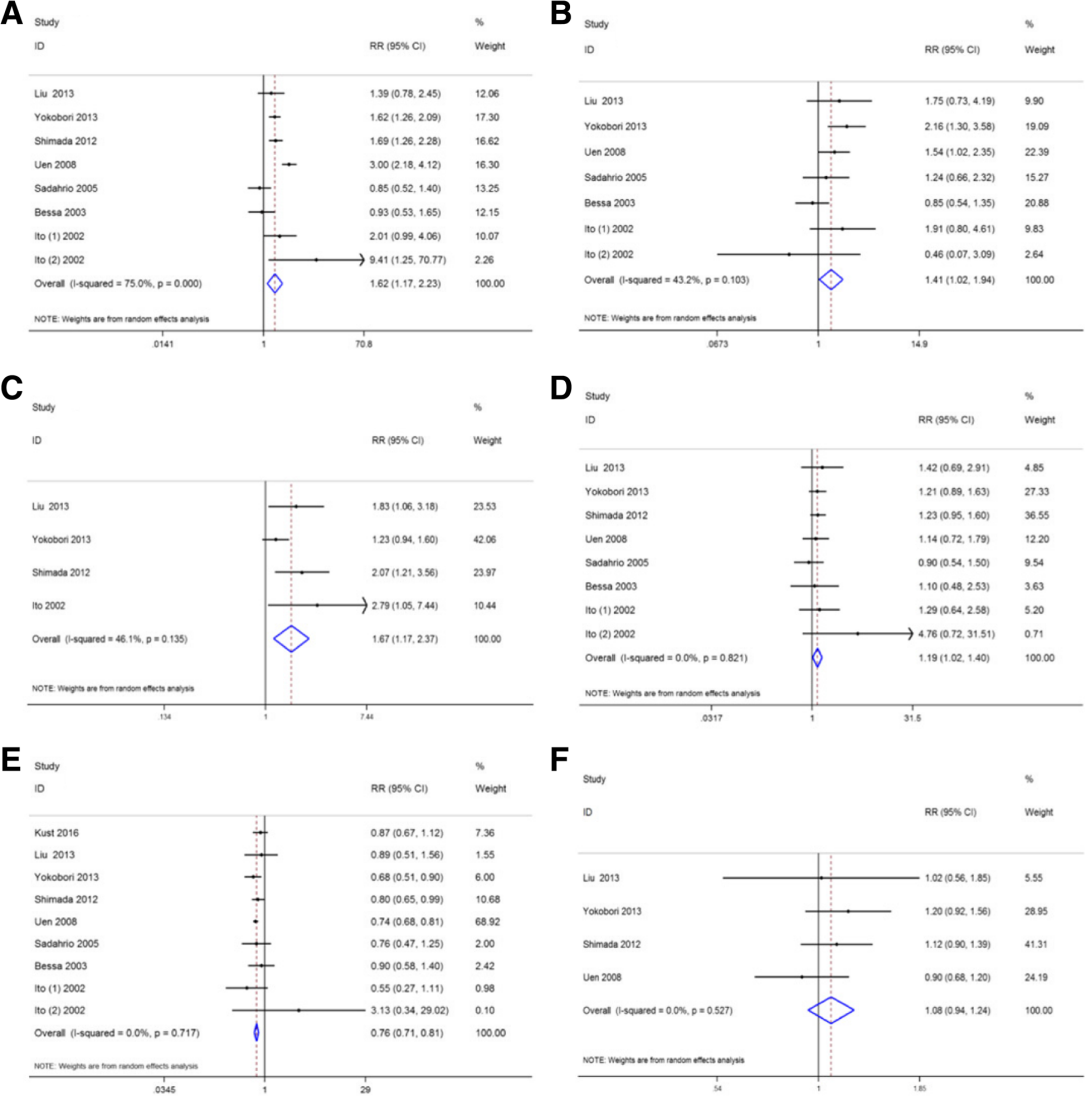
Summary estimates of risk ratio for clinicopathological parameters associated with CTCs-positive. **a** Regional lymph nodes metastasis; **b** Depth of infiltration; **c** Vascular invasion; **d** Tumor grade; **e** TNM stage (Stage I, II vs. Stage III); **f** Tumor size

Seven studies [19, 28, 29, 31, 35–37] including eight sets of data were evaluated to determine the relationship between CTC-positive and regional lymph nodes metastasis. The results showed regional lymph nodes metastasis was associated with CTC-positive (RR = 1.62, 95% CI: [1.17–2.23], *P* = 0.003) with significant between-study heterogeneity (I^2^ = 74.6%, *P*<0.001; Fig. 4a). The depth of tumor infiltration was associated with CTC-positive (RR = 1.41, 95% CI: [1.03–1.92], *P* = 0.03; I^2^ = 38.3%, *P* = 0.136; Fig. 4b). Studies assessed by pooled analysis showed significant association between CTC-positive and vascular invasion (RR = 1.66, 95% CI: [1.17–2.36], *P* = 0.004; I^2^ = 46.0%, *P* = 0.135; Fig. 4c). Eight sets of data from seven studies [19, 28, 29, 31, 35–37] demonstrated that tumor grade was associated with CTC-positive (RR = 1.19, 95% CI: [1.02–1.40], *P* = 0.029; I^2^ = 0%, *P* = 0.821; Fig. 4d). Eight studies [19, 20, 28, 29, 31, 35–37] reported the relationship between CTC-positive and TNM stage (I, II versus III). As shown in Fig. 3e, CTC-positive in stage III is greater than in stage I and II (RR = 0.76, 95% CI: [0.71–0.81], *P* < 0.001; I^2^ = 0%, *P* = 0.717; Fig. 4e). Furthermore, the pooled analysis found no significant relationship between CTC-positive and tumor size (RR = 1.08, 95% CI: [0.94–1.24], *P* = 0.30; I^2^ = 0%, *P* = 0.528; Fig. 4f).

### Exploring the sources of heterogeneity

To examine the intra-study inconsistencies on OS, we stratified the eligible studies according to variables as shown in Table 3. The pooled analyses results showed the heterogeneity did not drop to an insignificant level, regardless of the variables. Therefore, meta-regression was further implied to explore the source of heterogeneity on OS. As shown in Table 4, for the studies on OS, only positive rate of CTC detection was significantly correlated with intra-study variability (*P* = 0.021), and it explained 93.8% of the between-study variance in the multivariate analysis.

**Table 3:** Results of subgroup analyses on OS

Variables	HR[95%CI]	Number	I^2^	P^d^
Year > median^a^				
Yes	3.65[2.49–5.36]	6	0.00%	0.578
No	2.44[1.19–4.99]	7	72.90%	0.001
Country				
East Asia	3.39[2.27–5.05]	10	46.50%	0.051
Non-East Asia	2.10[0.52–8.54]	3	74.50%	0.02
Marker				
Single	2.72[1.48–5.00]	9	66.80%	0.002
Multiple	3.77[2.62–5.43]	4	0.00%	0.799
Sampling time point				
Pre-op	3.65[2.49–5.36]	6	0.00%	0.578
Intra/post-op	2.44[1.19–4.99]	7	72.90%	0.001
Patient no. > median^b^				
Yes	3.45[2.57–4.65]	6	0.00%	0.801
No	2.59[1.08–6.22]	7	74.20%	0.001
Detection rate > mean^c^				
Yes	1.57[0.42–5.79]	4	74.10%	0.009
No	3.71[2.84–4.85]	9	0.00%	0.796
Quality of study				
Low	4.06[1.64–10.05]	2	0.00%	0.384
High	2.95[1.87–4.65]	11	61.50%	0.004
Overall	3.07[2.05–4.62]	13	55.70%	0.008

NOTE: ^a^The median year for OS was 2012

^b^The median patient no. for OS was 103

^c^The mean detection rate for OS was 38.12%

^d^Two-tailed *P* value of tests for heterogeneity

**Table 4:** Results of meta-regression on OS

Variables	Coef.^a^	Std. Err.^b^	*P* value	Adj R-squared^c^
Year	0.5072	0.4559	0.2900	0.67%
Country	0.5352	0.5770	0.3740	1.08%
Marker	0.4133	0.5021	0.4280	−11.42%
Time point	−0.5072	0.4559	0.2900	0.67%
Patient no.	0.3751	0.4688	0.4410	−10.13%
Detection rate(mean)	−1.1526	0.4288	0.0210	93.80%
Quality of study	−0.3412	0.7123	0.6410	−12.93%

NOTE: ^a^Coef.: coefficient

^b^Std. Err.: standard Error

^c^Adj R-squared: Proportion of between-study variance explained

### Publication bias

**Fig. 5:**
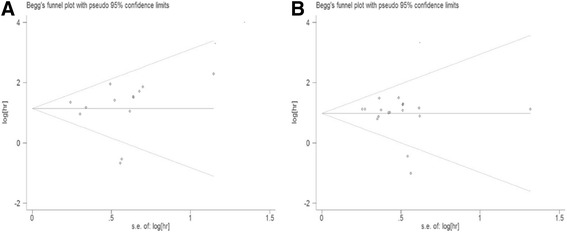
Publication bias analysis. **a** Funnel plot of the studies on overall survival; **b** Funnel plot of the studies on disease-free survival

Potential publication bias was assessed by Begg’s and Egger’s tests. *P* < 0.05 indicated the existence of publication bias. There was no evidence of publication bias for the pooled analysis of OS (*P*_Begg_ = 0.246, *P*_Egger_ = 0.964) and DFS (*P*_Begg_ = 0.434, *P*_Egger_ = 0.301). The funnel plots of publication bias on OS and DFS are shown in Fig. 5a and b, respectively.

## Discussion

Currently, the treatment strategies for non-mCRC include radical surgery as well as neoadjuvant and adjuvant radio-chemotherapy. In clinical practice, the oncologist selects the most appropriate regiment depending on the TNM stage, which is based on the extent of tumor invasion (T), the presence of metastases or micro-metastases in regional lymph nodes (N) and distant metastases (M) [38]. The clinical TNM stage, which is based on the imaging examination, can help oncologists assess whether neoadjuvant radio-chemotherapy should be incorporated before surgery, whereas the pathological TNM stage, which is based on the histopathologic examination of post-operative samples, provides information on whether adjuvant radio-chemotherapy should be included after curative resection. Despite advances in therapeutic approaches, it is estimated that approximately 30% of patients will develop metastases and eventually succumb to their disease after comprehensive treatment [39]. In general, the prognosis outcome of non-mCRC patients is directed by the TNM-stage, which provides the prognostic information with approximately 93% 5-year stage-specific survival rate for stage I, 84% for stage II, and 83% for stage III [40] and is influenced by clinicopathological parameters such as vascular invasion, poor differentiation, tumor size and serum tumor markers (i.e., carcinoembryonic antigen, CEA). Recently, many molecular biomarkers and high-risk gene signatures have been demonstrated to provide further information to support clinical decisions, however, none were conclusively accurate to evaluate the prognosis of all patients.

Since CTCs were first identified in PB of CRC patients, the clinical value of CTCs had become a debated topic throughout the medical community. From the clinical perspective, CTC analyses has an advantage in terms of a cost and ease of operation to serve as a monitoring tool pre and post treatments. Numerous studies had demonstrated that CTCs detection could provide important prognostic information for patients with CRC. A previous meta-analysis by Groot et al. had demonstrated the prognosis significance of detection of CTCs in patients with mCRC [41]. Peach et al. reviewed the prognostic value of postoperative detection of CTCs in non-mCRC patients and concluded that the presence of CTCs in PB was an independent predictor of recurrence [42]. However, the two meta-analyses were limited by the presence of methodological heterogeneity; the included studies used several different methods to detect CTCs and were not stratified by detection method. With regard to the detection methods of CTCs, the prognostic utility of the CellSearch system in CRC patients had been demonstrated by a meta-analysis [43]. However, the clinical application of the RT-PCR approach in the non-mCRC patients has still not been illustrated by a large-scale data analysis.

This study is the first comprehensive meta-analysis to validate the clinical significance of CTC detection by RT-PCR method only in non-mCRC. The results demonstrated that CTC-positive patients had poorer OS and DFS than CTCs-negative patients at different sampling time (pre-OP and intra/post-OP), TNM stage (II/III) and adjuvant therapy status (without/post-OP chemotherapy), indicating that the clinical prognosis of patients with non-mCRC is significantly associated with the CTCs detected by RT-PCR in PB. Our pooled analyses also assessed the association between CTCs and clinicopathological parameters of non-mCRC patients and showed that CTC-positive was correlated with regional lymph nodes metastasis, deep depth of tumor infiltration, vascular invasion, poor differentiation of tumor and later TNM stage. Moreover, all these parameters have been shown to be an indicators of poor prognosis in CRC patients. Combined with the results of our collective evaluation, CTC-positive in PB has been demonstrated to be considered a prognostic and predictive marker for patients with non-mCRC. Numerous studies have demonstrated that there was not relationship between tumor size and the positivity of CTCs detection [28, 35]; the results of our study were consistent with these previous studies.

Although we limited the studies included in our meta-analysis to those that used RT-PCR to reduce the heterogeneity caused by the difference in detection methods, no significant heterogeneity was found in the pooled analysis of DFS (I^2^ = 34.0%, *P* = 0.085). Nevertheless, there was still a certain extent of heterogeneity in our meta-analysis. Especially for OS, heterogeneity was mainly caused by data from the study by Shimada et al. [19]. Heterogeneity may also come from differences in the year, country and quality of publication, along with differences in sampling time, detection marker, or detection rate. Differences in the experimental designs in the cohort studies also generated non-negligible heterogeneity. To explore the potential sources of heterogeneity, subgroup analyses were performed based on year, country and quality of publication, sampling time, marker, number of patients, or detection rate, but the results were inconclusive (Table 3). Further, the results of the meta-regression clarified the heterogeneity and showed the detection rate was mainly responsible for the heterogeneity on OS. The detection rate of CTCs was greatly different based on different stage of early CRC. Stage I was too low, however, and the CTC-positive rate was significantly increased in stage III CRC patients, which had already been confirmed in studies using the CellSearch system [14, 15].

Theoretically, the association between prognosis and post-OP CTCs status was more convincing because post-OP CTCs status contains pre-OP CTCs and released CTCs during the operation [44]. However, the rapid apoptotic death of pre-OP CTCs may release mass tumor genes or antigens due to the change of the survival microenvironment in the process of operation, which might lead to a certain degree of detection bias. Therefore, the samples of post-OP samples could more accurately reflect the CTC status by including CTC release, apoptosis, and necrosis and could provide more information about the prognosis of patients. Ikeguchi M et al. [45] found that in blood samples collected within 48 h after the operation, patients with CTC-positive had better prognosis than CTCs-negative patients. In our meta-analysis, the estimated result for OS remained stable and was not significantly affected by sampling time, which indicated CTCs detection not only at pre-OP but also post-OP could provide a prognostic factor. Thus, uncertainties still remain that sampling time could provide more accurate prognostic information, and further studies are needed to evaluate this relationship.

There were several limitations in our meta-analysis. First, our data for the meta-analysis came from previously published studies, and several included studies did not report HR. Therefore, we had to calculate them from the reported data with limited access to the raw data, which might affect the accuracy of the results. Second, there was considerable heterogeneity in our study. Although we eliminated the heterogeneity from detection methodology, RT-PCR cannot achieve CTCs enumeration and lacks biologic specificity. However, it does have the advantage of high sensitivity for CTCs detection [46]. We addressed the between-study heterogeneity by using a random effects model to obtain more conservative estimates. Third, language selection brings bias. We restricted the eligible studies to those written in English and excluded the relevant studies of other languages according to language criteria, which may cause language bias leading to an overestimation of effect sizes [47]. Despite these limitations, our meta-analysis is the first study to assess the prognostic significance of CTCs detected by RT-PCR in non-mCRC patients. Our results provides an example for other studies that standardized testing method, optimized sampling time, complete analysis and report of results should be used to derive more accurate prognostic significance of CTCs in non-mCRC and CRC patients.

## Conclusions

Based on available evidence, our meta-analysis suggested that the detection of CTCs in PB by RT-PCR is a prognostic factor for patients with non-mCRC, and CTC-positive was associated with poor prognosis and poor clinicopathological prognostic factors. However, the prognostic value of CTCs supports the use of CTCs as an indicator of metastatic disease prior to the current classification of mCRC meaning it is detectable by CT/MRI. Further high-quality, well-designed, large-scale multicenter studies are required to evaluate the clinical significance and utility of CTCs detected by RT-PCR in non-mCRC patients.

## Abbreviations

CIs: Confidence intervals
CRC: Colorectal cancer
CT: Chemotherapy
CTCs: Circulating tumor cells
DFS: Disease-free survival
HR: Hazard ratio
mCRC: Metastatic CRC
non-mCRC: Non-metastatic CRC
OP: Operative
OS: Overall survival
PB: Peripheral blood
RLNs: Regional lymph nodes
RR: Relative risk
RT-PCR: Reverse-transcriptase polymerase chain reaction
TNM: Tumor-node-metastasis
